# Skeletal Muscle Strength in Patients on the Heart Transplant Waiting List: Are There Any Associations with Instrumental Examination Data?

**DOI:** 10.3390/jcm15020665

**Published:** 2026-01-14

**Authors:** Alexey N. Sumin, Anna V. Shcheglova, Darya P. Golubovskaya, Yaroslav I. Bryukhanov, Darina N. Fedorova, Maria I. Anchkova, Tamara B. Pecherina

**Affiliations:** Federal State Budgetary Scientific Institution “Research Institute for Complex Issues of Cardiovascular Diseases”, Academician L. S. Barbarash Boulevard, Building 6, 650002 Kemerovo, Russia; nura.karpovitch@yandex.ru (A.V.S.); goludp@kemcardio.ru (D.P.G.); bryuyi@kemcardio.ru (Y.I.B.); fedorova.darina.2001@mail.ru (D.N.F.); masha.anchkova@mail.ru (M.I.A.); tb.pechorina@gmail.com (T.B.P.)

**Keywords:** heart transplant, chronic heart failure, muscle status, echocardiography, volumetric sphygmography

## Abstract

**Background**: Peripheral muscle dysfunction in chronic heart failure (CHF) potentiates hemodynamic insufficiency through neuroendocrine activation and deterioration of myocardial perfusion. **Objectives**: The aim of this study was to compare skeletal muscle strength in patients on the heart transplant (HT) waiting list and in patients undergoing cardiac surgery and to identify associations between muscle status and clinical and instrumental parameters. **Methods**: This study included 152 patients divided into two groups: Group I (*n* = 30)—candidates for HT; Group II (*n* = 122)—patients undergoing cardiac surgery. A comprehensive clinical and anamnestic assessment and instrumental diagnostics were performed. Muscle status was assessed using a Lafayette MMT01165 isokinetic dynamometer (knee and foot extensor/flexor strength) and a DK 100 dynamometer (handgrip strength). Echocardiographic parameters were assessed in the study groups; in the HT group, cardiac catheterization and volumetric sphygmography data were used. **Results**: Group HT had a higher proportion of men (*p* = 0.042) and a higher revascularization rate (*p* ≤ 0.027) compared with Group II. Patients in Group I had enlarged left and right heart diameters and critically low left ventricular ejection fraction (LVEF) (22%; *p* < 0.001). Group HT showed a significant decrease in lower extremity muscle strength compared with Group II (*p* < 0.001). The only independent predictors of knee extensor muscle strength were echocardiographic parameters (right atrial volume index, TAPSE, and LVEF/end diastolic volume). No association was found between knee extensor strength and the strength of other muscle groups studied. **Conclusions**: Patients on the HT waiting list had significant lower extremity muscle weakness compared with preoperative patients. The only independent predictors of knee extensor muscle strength were echocardiographic parameters. Knee extensor muscle strength was associated only with contralateral muscle strength, but not with other muscle groups. These results will facilitate the development and evaluation of personalized rehabilitation programs for patients on the heart transplant waiting list.

## 1. Introduction

Chronic heart failure (CHF) typically results from primary cardiac damage, which impairs myocardial function and leads to central hemodynamic impairment. Furthermore, patients with CHF experience changes in skeletal muscle, and this peripheral dysfunction exacerbates central hemodynamic impairment. This phenomenon has been termed the “muscle hypothesis,” which explains the development of CHF symptoms [1]. It has been shown that in CHF, increased pressor ergoreflex response to exercise leads to excessive increases in heart rate, pulmonary ventilation, sympathetic nerve activity, and mean arterial pressure [2]. Sensitization of this reflex can lead to increased dyspnea during exercise and, consequently, decreased exercise tolerance. Furthermore, sympathetic activation due to increased ergoreflex reduces the effectiveness of modern drug therapy for CHF and, consequently, accelerates disease progression [1].

It is not surprising that skeletal muscle dysfunction is most pronounced in patients with severe CHF, reaching the stage of sarcopenia. These patients are candidates for left ventricular assist devices or for inclusion on the heart transplantation (HT) waiting list [3]. Indeed, HT is the gold standard in the treatment of this category of patients, allowing for a radical improvement in the pumping function of the recipient’s heart [4]. However, after successful HT, not only the functional status but also the prognosis of patients depends on the degree of muscle dysfunction [3]. After successful HT, muscle status gradually improves due to increased physical activity, especially during physical rehabilitation [3,5,6]. However, one cannot rely solely on the effect of rehabilitation measures after a successful HT. Due to the current shortage of donor hearts, patients can spend considerable time on the waiting list, during which time muscle maladaptation becomes most pronounced, complicating subsequent postoperative rehabilitation. Furthermore, in patients on the waiting list, skeletal muscle condition is a prognostic factor for emergency left ventricular assist device (LVAD) implantation or death [7,8]. Therefore, interventions aimed at preventing/reducing the progression of muscle dysfunction in patients on the HT waiting list appear appropriate. Currently, there are a few studies using carefully selected physical training programs for this category of patients [9,10], but such programs face difficulties in translating into real-world clinical practice (poor exercise tolerance, inability to attend rehabilitation centers, etc.). Therefore, it seems logical to use local training effects, primarily on skeletal muscles, without systemic effects on hemodynamics. For this purpose, it is possible to employ electrical muscle stimulation (EMS) of skeletal muscles, which is used in patients with critical illnesses [11], including in cardiac surgery [12]. This method can be safely used both in complicated postoperative periods [13] and in prehabilitation before cardiac surgery [14]. Therefore, studies of skeletal muscle condition in patients with CHF on the waiting list for heart transplantation deserve special attention. The above served as the basis for this study, the aim of which was to compare skeletal muscle strength in patients on the waiting list for heart transplantation and in patients before cardiac surgery, as well as to identify associations between muscle status and clinical and instrumental indicators.

## 2. Material and Methods

### 2.1. Study Group Characteristics

The study was conducted at the Research Institute for Complex Issues of Cardiovascular Diseases (Kemerovo, Russia). The study analyzed two cohorts of patients with different recruitment designs and time frames. The prospective cohort included patients with CHF who were either already included in the HT waitlist or were considered candidates for inclusion in the waitlist (*n* = 30, 26 men and 4 women, mean age 58.3 ± 9.6 years; group 1). The retrospective cohort included patients undergoing cardiac surgery before planned cardiac intervention (*n* = 122, 83 men and 39 women, mean age 62.8 ± 5.0 years; group 2) in the period from 7 September 2020, to 30 September 2022. The total sample size was 155 patients. Inclusion criteria for all groups included signed informed consent and no acute medical conditions in the past 3 months (for the first group, a confirmed diagnosis of CHF with a corresponding indication for expected heart transplantation; for the second group, preparation for cardiac surgery with cardiopulmonary bypass). Exclusion criteria for both groups included cancer, severe neurological disorders, musculoskeletal defects, life-threatening rhythm/conduction disturbances, unstable hemodynamics, clinically significant pulmonary hypertension, decreased or lost cognitive function that prevented full compliance with the study protocol, emergency or urgent surgical interventions, and refusal to participate. Patients underwent a standard examination, including demographic, clinical, and instrumental studies. Anamnestic parameters included the presence of myocardial revascularization, myocardial infarction, stroke, arterial hypertension, and diabetes mellitus.

The study was conducted in accordance with the Declaration of Helsinki and approved by the local ethics committee of the Research Institute of Cardiovascular Surgery (protocol No. 5 dated 7 April 2025) with the support of the Russian Science Foundation grant No. 25-15-20054 “Methodology of physical prehabilitation in the form of passive physical training using EMS in patients with severe heart failure from the waiting list for orthotopic heart transplantation”.

### 2.2. Data Collection

#### 2.2.1. Echocardiography

Echocardiography was performed on a Vivid 7Dimension system (General Electric, Boston, MA, USA) for a comprehensive assessment of the structural and functional parameters of the heart in accordance with current guidelines [15]. The following parameters were assessed in all patients: left ventricular diameter and wall thickness, and aortic dimensions were measured in two-dimensional M-mode. Left ventricular end-systolic and end-diastolic volumes, left ventricular myocardial mass, left atrial transverse diameter (LA) in diastole, and the ratio of early to late transmitral diastolic flow (E/A) were also assessed. Left ventricular ejection fraction (LVEF) was calculated using Simpson’s method. Additionally, the left atrial volume index, right atrial volume index, left ventricular myocardial mass index, and TAPSE were assessed in the HT group.

#### 2.2.2. Skeletal Muscle Strength Assessment

Muscle status was assessed by measuring the strength of the knee extensors and flexors, as well as the foot flexors and extensors, using a Lafayette MMT01165 isokinetic dynamometer (Lafayette Instrument Company, Lafayette, IN, USA). Measurements were taken in a sitting position at maximum muscular effort; the results (maximum muscle strength) were recorded in real time on the device’s built-in screen. Four paired exercises were performed for each muscle group. Grip strength was determined using a DK 100 dynamometer (Nizhny Tagil Medical Instrument Plant, Nizhnij Tagil, Russia) by sequentially measuring the right and left hands and recording paired readings. All procedures were performed according to a standardized protocol to ensure reproducibility of the results.

#### 2.2.3. Arterial Wall Stiffness Assessment

Patients in the HT group with severe CHF additionally underwent hemodynamic measurements using volumetric sphygmography on a VaSeraVS-1000 device (Fukuda Denshi, Tokyo, Japan). Cuffs were applied to the arms and legs, ECG signals and a phonocardiogram were recorded, and systolic and diastolic blood pressure (SBP/DBP), pulse pressure (PAP), cardio-ankle vascular index (CAVI), and ankle-brachial index (ABI) were determined.

#### 2.2.4. Heart Catheterization

During right heart catheterization, the following hemodynamic parameters were recorded: pulmonary artery pressure (PAP), including systolic and mean; pulmonary capillary wedge pressure; pulmonary vascular resistance; and transpulmonary gradient. Cardiac output and index were also assessed using thermodilution.

### 2.3. Statistical Analysis

Statistical data processing was performed using the SPSS Statistics 17.0.1 (IBM, Armonk, NY, USA). software package. Quantitative indicators are presented as medians with lower and upper quartiles (Me [LQ;UQ]). Categorical variables are expressed as absolute values and percentages. The nonparametric Mann–Whitney test was used to compare quantitative indicators between groups; the Pearson chi-square test was used for categorical variables. The association between right and left knee extensor muscle strength was assessed using linear regression analysis in two models: (1) with instrumental indicators (echocardiography, cardiac catheterization, and volumetric sphygmography data) and (2) with the strength of other muscle groups. Differences were considered statistically significant at *p* < 0.05.

## 3. Results

Analysis of demographic and clinical anamnestic data revealed significant differences between the groups (Table 1). Group I (HT candidates) had a higher proportion of men (*p* = 0.042), higher body mass index (*p* < 0.05), and higher revascularization rate (*p* = 0.001) compared to Group II. There were no differences in age, hypertension, or history of myocardial infarction between the groups (*p* > 0.05).

Analysis of echocardiographic parameters revealed statistically significant differences between the groups (Table 1). Patients in Group I (candidates for HT) showed significant structural and functional changes: a significant increase in left atrial size (*p* < 0.001), end-systolic (<0.001) and end-diastolic (*p* < 0.01) LV volumes with critically low LVEF (22.0%; *p* < 0.001). Diastolic dysfunction, assessed by the ratio of the maximum left ventricular early diastolic filling velocity (peak E) to the maximum late atrial filling velocity (peak A) with transmitral blood flow E/A, was significantly more pronounced in patients in Group I compared to Group II (*p* < 0.001), reflecting the severity of the condition in terminal CHF.

Analysis of muscle status revealed significant weakness of the skeletal muscles of the lower extremities in patients in the HT group compared to patients before cardiac surgery (Table 2). The knee extensor and flexor strength in Group I was 40–50% lower than in Group II (*p* < 0.001 in all cases).

The results of invasive cardiac catheterization in patients on the HT waiting list are presented in Table 3. The data indicate a moderate increase in pressure in the pulmonary circulation, as well as the absence of signs of precapillary pulmonary hypertension, which is consistent with the picture of systolic-diastolic dysfunction in CHF. Also, in this group, analysis of volumetric sphygmography data revealed the following blood pressure levels: median SBP was 125.0 (116.0; 136.0) mmHg, DBP—78.0 (72.0; 86.0) mmHg, PBP—44.0 (32.0; 53.0) mmHg. The median CAVI reached 8.5 (6.5; 9.7), indicating increased vascular stiffness. The median ABI was near the lower limit of normal—0.96 (0.8; 1.0), which may also indicate subclinical damage to the peripheral vascular bed.

### Regression Analysis Results

Multiple linear regression was used to assess the association of lower limb muscle strength with clinical and instrumental parameters (age, weight, echocardiography data, cardiac catheterization, vascular stiffness assessment results).

Right knee extensor muscle strength (Table 4) was associated with left atrial diameter (β = 0.625; *p* = 0.02), interventricular septal thickness (β = 14.706; *p* = 0.001), posterior wall thickness (β = −15.181; *p* = 0.001); aortic dimensions (β = −0.793; *p* = 0.002); left atrial volume index (β = −1.657; *p* = 0.004); TAPSE (β = −0.669; *p* = 0.001); pulmonary artery pressure, mean (β = 2.124; *p* = 0.001); pulmonary capillary wedge pressure (β = −1.903; *p* = 0.001); CAVI on the right (β = −2.587; *p* = 0.001) and left (β = 2.130; *p* = 0.001). In stepwise linear regression, only echocardiographic parameters (right atrial volume index, TAPSE, and LVEF) were independent predictors of right knee extensor muscle strength.

Left knee extensor muscle strength (Table 5) was associated with interventricular septal thickness (β = 9.050; *p* = 0.006), posterior wall thickness (β = −9.235; *p* = 0.007); aortic size (β = −0.454; *p* = 0.026); left atrial volume index (β = −1.296; *p* = 0.011); TAPSE (β = −0.533; *p* = 0.003); CAVI on the right (β = −2.257; *p* = 0.002) and left (β = 2.037; *p* = 0.002); pulmonary artery pressure, mean (β = 2.244; *p* < 0.001) and pulmonary capillary wedge pressure (β = −1.785; *p* < 0.001). In stepwise linear regression (Table 6), echocardiographic parameters of the right (Right atrial volume index, TAPSE) and left (LV End Diastolic Volume) heart were independent predictors of left knee extensor muscle strength.

In the analysis of the second model of linear regression analysis, it was revealed (Table 6) that the strength of the right knee extensor muscles in univariate analysis did not reveal a significant association with the strength of other muscles, except for the strength of the left knee extensor muscles (β = 0.653, *p* = 0.052). At the same time, in the stepwise regression analysis, the association between these indicators was more significant (β = 0.750, *p* = 0.001). In stepwise regression analysis (Table 7), the strength of the left knee extensor muscles was associated with right knee extensor strength (β = 0.550, *p* = 0.001), right handgrip strength (β = 0.358, *p* = 0.01), and right ankle flexor strength (β = 0.296, *p* = 0.048).

## 4. Discussion

This study demonstrated that patients with CHF on the heart transplantation waiting list had a significant decrease in lower extremity skeletal muscle strength compared to patients examined before elective cardiac surgery. In addition, factors associated with skeletal muscle strength were assessed in the group of patients on the heart transplantation waiting list using linear regression analysis. In univariate analysis, echocardiographic parameters of the left and right heart, invasive hemodynamic study data, and the CAVI (a measure of arterial wall stiffness) were associated with knee extensor strength. However, in multiple regression analysis, only echocardiographic parameters of the right heart (TAPSE and RAVind) and one left ventricular parameter (EF or EDV) were independently associated. However, univariate regression analysis revealed no significant association between knee extensor strength and the strength of the other muscle groups studied. However, these associations approach statistical significance, and stepwise models reveal contralateral and individual muscle relationships. A possible explanation for these findings is the asymmetry and specificity of muscle groups.

In patients on the HT waiting list, other issues are traditionally examined when assessing muscle status, such as associations with functional indicators (physical performance using spiroergometry or a six-minute walk test) or with patient prognosis [16,17]. For example, preoperative sarcopenia, diagnosed using the pectoralis area index, was an independent predictor of survival and discharge from hospital (DAOH) after heart transplantation [16]. Patients with sarcopenia had a longer index hospitalization and were more likely to be discharged to a health facility other than home. In a Poisson regression model, sarcopenia was a significant univariate and the strongest multivariate predictor of DAOH at 1 year [16]. Accordingly, physical training programs for patients after HT have been shown to improve muscle status. Traditional endurance or strength training regimens performed for 3–6 months can normalize at least part of the skeletal muscle deficit after HT by increasing muscle mass and strength consistent with improvements in mitochondrial and type I oxidative fiber function/morphology [3,5].

Of particular interest are the causes of sarcopenia development after HT. In a cross-sectional study, multivariate analysis identified the following significant predictors of sarcopenia: diabetes, functional capacity (with a nonlinear relationship), and kinesiophobia [18]. These results highlight the importance of appropriate patient management to prevent sarcopenia after HT, including while patients are on the HT waiting list.

The results obtained in this study on factors associated with muscle strength in patients on the waiting list for HT are interesting in themselves. However, the greater clinical significance of these data lies in their subsequent use in prehabilitation programs or electrical muscle stimulation. Indeed, little attention is currently paid to assessing and correcting muscle status in patients on the HT waiting list. However, it is known that the risk of emergency LVAD implantation/death on the HT waiting list decreased by 10% for each increase in muscle area per cm^2^/m^2^ (OR 0.901; *p* = 0.049) [7]. To date, there are virtually no studies on physical training in such patients. Multimodal rehabilitation in patients awaiting heart transplantation has been shown to improve skeletal muscle function (an increase in the Medical Research Council score from 52.0 ± 7 to 58.7 ± 3; *p* = 0.042) [10]. However, this type of rehabilitation is not suitable for all patients on the waiting list, who are typically observed outside of hospitals. Outpatient training is possible for selected motivated patients. For example, a clinical case of long-term training (up to 5 years) in a patient on the HT waiting list is described. The training regimen in this case was characterized by a cardiopulmonary test every 4 weeks with subsequent adjustment of the training intensity. Each cardiac rehabilitation session consisted of a 30-min exercise bike workout at 100% of the anaerobic threshold, a 30-min course of wellness therapy, and 30 min of stretching exercises. These sessions were five per week, supplemented by 60 min of walking [9]. However, implementing such a rehabilitation program for a significant number of patients in even one center has not yet been possible.

Currently, it appears that carefully selected, individualized training programs are feasible for patients with severe CHF, effectively improving their muscular status. For example, in patients with functional class III CHF, training tailored to their lactate threshold [19] has been shown to have a positive effect on skeletal muscle. Following physical rehabilitation, improvements in skeletal muscle morphofunctional parameters were observed in repeat calf muscle biopsies: a decrease in muscle fiber diameter, an increase in alkaline phosphatase activity, and a decrease in lactate dehydrogenase activity in muscle fibers. These dynamics may indicate improved muscle blood flow and demonstrate the realization of regenerative potential in muscle fiber precursor cells, as confirmed by morphological and histochemical studies [19]. It remains unclear how effective this training program will be in patients on the HT waiting list.

Some patients on the HT waiting list receive LVAD implantation as part of their therapy. In this particular patient category, assessment and intervention of their muscle status are also relevant. For example, in patients with CHF with high baseline muscle atrophy, a significant increase in skeletal muscle mass was observed during the first 6 months of LVAD support [20]. For example, implantation of a mechanical circulatory support device allows for the activation of patients: potential reversibility of impairments in muscle function 2–6 months after implantation has been demonstrated, even without an exercise program, with adequate treatment of heart failure [21]. However, the results of recent meta-analyses have been contradictory. According to one of them (based on an analysis of 12 studies), cardiac rehabilitation after implantation of a left ventricular assist device was shown to be accompanied by a more pronounced improvement in the functional capacity of patients (improvement in VO2max and 6-min walk test distance) compared to standard therapy [22]. At the same time, another similar meta-analysis found no effect of cardiac rehabilitation on these parameters in this patient population [23]. Moreover, the increase in muscle mass during the first 6 months after LVAD implantation was most closely associated with improved neurohumoral stability in CHF and inflammation (i.e., decreased NT-proBNP and hsCR levels), rather than with daily activity levels or daily protein intake [24]. As we can see, even LVAD implantation does not solve many problems in patients on the HTx waiting list.

Therefore, targeted interventions for skeletal muscle status using EMS appear quite relevant. For example, in a recent study, the use of EMS in critically ill patients prevented a decrease in quadriceps size and also contributed to an increase in muscle strength as measured by the Medical Research Council scale [11]. A recent systematic review also presents the results of using EMS in cardiac surgery patients [12]. This analysis presents the results of nine studies using EMS after cardiac surgery and only one study using it before surgery. Most studies failed to demonstrate an improvement in overall functional capacity, but such training was safe and led to improvements in skeletal muscle strength and function. These results were consistent with the above data on the dynamics of these parameters after LVAD implantation. These studies used stimulation of various muscle groups of the lower extremities—quadriceps muscle, vastus medialis and lateralis muscle, triceps surae muscle, and gastrocnemius muscle—in various combinations [12]. As our study showed, the strength of individual muscles of the lower extremities did not correlate with each other, so to evaluate the effectiveness of EMS courses, it makes no sense to evaluate the dynamics of global muscle strength (for example, according to the Medical Research Council scale as in the study by Lorenzoni BS et al. [11]. In this case, the real effect of the EMS course can be underestimated, so it is necessary to study the strength of only those muscles that were exposed.

When evaluating the study results, its limitations should be considered. First, it was conducted at a single center, so our data should be applied with caution to other centers. Second, the group of patients before HT was relatively small; however, given the limited number of donor hearts available per year, adding a larger number to the waiting list does not make sense. Third, we assessed only one indicator of muscle status—strength. A number of studies have assessed other indicators, such as muscle mass using computed tomography [7] or ultrasound [25]. However, our method of assessing muscle strength using portable dynamometers is more suitable for studying the dynamics of muscle status, as it does not require specialized equipment. It should be taken into account that in this study we did not evaluate the level of biomarkers (in particular, troponin T and NT-proBNP) and could not assess their relationship with muscle condition in the groups, which is a limitation of the study. Furthermore, muscle status was assessed at different times in the HT and comparison groups. Nevertheless, there is no reason to assume that muscle status could have changed significantly in the studied groups over time. Since the number of predictors relative to the sample size in the HT group is quite large, this may lead to potential overfitting. Therefore, the results obtained in the regression models should be considered preliminary rather than definitive.

## 5. Conclusions

Patients on the heart transplant waiting list exhibit a significant decrease in lower extremity muscle strength compared to patients undergoing cardiac surgery. Multiple regression analysis revealed that only right heart echocardiographic parameters and one left ventricular parameter (EF or EDV) were independently associated with extensor muscle strength. However, univariate regression revealed no significant association between knee extensor strength and the strength of the other muscle groups studied. These results will facilitate the development and evaluation of personalized skeletal EMS programs for patients on the heart transplant waiting list.

## Figures and Tables

**Table 1:** Demographic and clinical–anamnestic characteristics of the study groups.

Indicators *n*, %; Me [LQ; UQ]	Group I HT Waiting List (*n* = 30)	Group II Before Cardiac Surgery (*n* = 122)	*p*
Men (*n*, %)	26 (86.7)	83 (68.0)	0.042
Age (years)	61.5 [51.0; 65.0]	63.0 [58.5; 68.0]	>0.100
BMI (kg/m^2^)	29.5 [25.9; 31.4]	28.0 [25.7; 32.4]	<0.05
Myocardial infarction history (*n*, %)	15 (50.0)	61 (50.0)	1.00
Hypertension (*n*, %)	28 (93.3)	100 (82.0)	0.126
Stroke history (*n*, %)	1 (3.3)	13 (10.7)	0.214
Diabetes history (*n*, %)	8 (26.7)	37 (30.3%)	0.694
History of revascularization (*n*, %)	21 (70.0)	13 (10.7%)	0.001
Left Atrial Diameter (cm)	5.2 [4.6; 5.8]	4.55 [4.2; 5.2]	<0.001
LV End-Systolic Volume (mL)	150.0 [140.0; 239.0]	69.0 [47.0; 113.0]	<0.001
LV End-Diastolic Volume (mL)	202.0 [181.0; 290.0]	176.5 [135.0; 231.0]	<0.01
Interventricular Septal Thickness (mm)	1.0 [0.9; 1.2]	1.0 [1.0; 1.3]	>0.100
Posterior wall thickness (mm)	1.0 [0.8; 1.2]	1.0 [1.0; 1.2]	>0.100
Aorta (cm)	3.5 [3.3; 4.0]	3.5 [3.3; 3.8]	>0.100
LV ejection fraction (%)	22.0 [18.0; 31.0]	61.5 [48.0; 67.0]	<0.001
E/A ratio	1.2 [0.6; 3.2]	0.73 [0.62; 1.06]	<0.001

Notes: BMI—Body Mass Index, HT—heart transplant, LV—left ventricular.

**Table 2:** Muscle status in patients on the heart transplant waiting list and in patients before cardiac surgery.

Indicators *n*, %; Me [LQ; UQ]	Group I HT Waiting List (*n* = 30)	Group II Before Cardiac Surgery (*n* = 122)	*p*
Right knee extensor strength (kg)	17.05 [12.25; 19.95]	24.6 [19.2; 32.3]	<0.001
Left knee extensor strength (kg)	14.6 [10.8; 19.4]	24.8 [19.3; 31.3]	<0.001
Right knee flexor strength (kg)	12.2 [8.7; 14.85]	19.3 [13.2; 25.0]	<0.001
Left knee flexor strength (kg)	11.9 [9.75; 14.25]	19.3 [13.2; 25.0]	<0.001
Right ankle extensor strength (kg)	9.0 [6.0; 12.5]	30.6 [24.9; 35.1]	<0.001
Left ankle extensor strength (kg)	8.7 [7.4; 11.5]	28.9 [24.0; 34.7]	<0.001
Right ankle flexor strength (kg)	11.0 [8.4; 16.5]	20.7 [15.8; 25.7]	<0.001
Left ankle flexor strength (kg)	12.0 [8.2; 13.4]	20.3 [16.3; 24.4]	<0.001
Right handgrip strength (kg)	32.1 [21.0; 41.0]	28.8 [19.8; 34.0]	>0.100
Left handgrip strength (kg)	28.0 [22.0; 39.0]	24.5 [16.5; 31.0]	>0.050

**Table 3:** Results cardiac catheterization and assessment of arterial wall stiffness in patients on the heart transplant waiting list.

Indicators	Me [LQ; UQ]
Cardiac catheterization	
Pulmonary artery pressure, systolic (mmHg)	24.0 [22.0; 40.0]
Pulmonary artery pressure, mean (mmHg)	29.0 [22.0; 40.0]
Pulmonary capillary wedge pressure (mmHg)	20.0 [14.0; 26.0]
Cardiac index (L/min/m^2^)	2.1 [1.8; 2.4]
Cardiac output (L/min)	4.3 [3.35; 5.0]
Transpulmonary gradient (mmHg)	9.0 [7.0; 11.0]
Pulmonary vascular resistance (Wood’s unit)	1.8 [1.4; 2.8]
Vascular stiffness and hemodynamic indicators	
Systolic blood pressure (mmHg)	125.0 [116.0; 136.0]
Diastolic blood pressure (mmHg)	78.0 [72.0; 86.0]
Pulse arterial pressure (mmHg)	44.0 [32.0; 53.0]
Heart rate (beats/min)	72.0 [65.0; 85.0]
Cardio-ankle vascular index	8.5 [6.5; 9.7]
Ankle-brachial index	0.96 [0.8; 1.0]

**Table 4:** Association of the strength of the right knee extensors with instrumental indicators (linear regression analysis, Enter method + Stepwise method).

Indicators	B	Std. Error	Beta	t	Sig.
Enter method					
(Constant)	82.049	14.191		5.782	0.000
Age (years)	−0.030	0.058	−0.058	−0.517	0.615
Weight (kg)	−0.014	0.041	−0.058	−0.352	0.732
LV End-Diastolic Volume (mL)	0.109	0.057	1.907	1.919	0.081
LV End-Systolic Volume (mL)	−0.030	0.061	−0.495	−0.489	0.634
Left Atrial Diameter (cm)	5.009	1.834	0.625	2.731	0.020
Interventricular Septal Thickness (mm)	189.857	37.307	14.706	5.089	0.000
Posterior wall thickness (mm)	−223.779	44.793	−15.181	−4.996	0.000
Aorta (cm)	−10.860	2.638	−0.793	−4.117	0.002
LV ejection fraction (%)	0.373	0.281	0.832	1.329	0.211
Left atrial volume index (mL/m^2^)	−0.640	0.177	−1.657	−3.614	0.004
Right atrial volume index (mL/m^2^)	0.162	0.125	0.386	1.292	0.223
Left ventricular myocardial mass index (g/m^2^)	0.030	0.061	0.344	0.498	0.628
TAPSE (mm)	−9.121	2.043	−0.669	−4.465	0.001
Cardio-ankle vascular index (right)	−5.347	1.251	−2.587	−4.274	0.001
Cardio-ankle vascular index (left)	4.685	1.178	2.130	3.978	0.002
Pulmonary artery pressure, mean (mmHg)	1.008	0.184	2.124	5.478	0.000
Pulmonary capillary wedge pressure (mmHg)	−1.117	0.192	−1.903	−5.826	0.000
Cardiac index (L/min/m^2^)	−3.092	2.922	−0.272	−1.058	0.313
Cardiac output (L/min)	−0.037	1.521	−0.007	−0.024	0.981
Transpulmonary gradient (mmHg)	−0.660	0.478	−0.571	−1.382	0.195
Pulmonary vascular resistance (WU)	−1.215	1.391	−0.366	−0.874	0.401
Stepwise method					
(Constant)	42.835	4.364		9.815	0.000
Right atrial volume index mL/m^2^	−0.300	0.055	−0.713	−5.420	0.000
TAPSE (mm)	−6.072	1.777	−0.445	−3.416	0.002
LV ejection fraction (%)	−0.165	0.059	−0.367	−2.787	0.009

Notes: LV—left ventricular, TAPSE—systolic excursion of the tricuspid annulus plane.

**Table 5:** Association of the strength of the left knee extensors with instrumental indicators (linear regression analysis, Enter method + Stepwise method).

Indicators	B	Std. Error	Beta	t	Sig.
Enter method					
(Constant)	52.672	13.696		3.846	0.003
Age (years)	−0.034	0.056	−0.064	−0.620	0.548
Weight (kg)	0.000	0.039	−0.003	−0.020	0.984
LV End-Diastolic Volume (mL)	0.098	0.055	1.641	1.796	0.100
LV End-Systolic Volume (mL)	−0.018	0.059	−0.290	−0.312	0.761
Left Atrial Diameter (cm)	2.966	1.770	0.353	1.676	0.122
Interventricular Septal Thickness (mm)	122.674	36.007	9.050	3.407	0.006
Posterior wall thickness (mm)	−142.932	43.231	−9.235	−3.306	0.007
Aorta (cm)	−6.525	2.546	−0.454	−2.563	0.026
LV ejection fraction (%)	0.382	0.271	0.810	1.408	0.187
Left atrial volume index (mL/m^2^)	−0.526	0.171	−1.296	−3.076	0.011
Right atrial volume index (mL/m^2^)	0.058	0.121	0.133	0.483	0.639
Left ventricular myocardial mass index (g/m^2^)	0.011	0.059	0.123	0.194	0.850
TAPSE (mm)	−7.640	1.972	−0.533	−3.875	0.003
Cardio-ankle vascular index (right)	−4.898	1.208	−2.257	−4.056	0.002
Cardio-ankle vascular index (left)	4.704	1.137	2.037	4.139	0.002
Pulmonary artery pressure, mean (mmHg)	1.118	0.178	2.244	6.297	0.000
Pulmonary capillary wedge pressure (mmHg)	−1.101	0.185	−1.785	−5.946	0.000
Cardiac index (L/min/m^2^)	0.255	2.820	0.021	0.091	0.929
Cardiac output (L/min)	−1.263	1.468	−0.244	−0.860	0.408
Transpulmonary gradient (mmHg)	−0.789	0.461	−0.650	−1.711	0.115
Pulmonary vascular resistance (WU)	−1.811	1.342	−0.519	−1.349	0.204
Stepwise method					
(Constant)	35.640	4.622		7.711	0.000
Right atrial volume index (mL/m^2^)	−0.336	0.058	−0.763	−5.780	0.000
TAPSE (mm)	−6.756	1.823	−0.472	−3.707	0.001
LV End-Diastolic Volume (ml)	0.016	0.008	0.272	2.160	0.039

Notes: LV—left ventricular, TAPSE—systolic excursion of the tricuspid annulus plane.

**Table 6:** Association of the strength of the right knee extensor muscles with the strength of other muscle groups (linear regression analysis, Enter method + stepwise method).

Indicators	B	Std. Error	Beta	t	Sig.
Enter method					
(Constant)	4.269	6.628		0.644	0.534
Left knee extensor strength (kg)	0.622	0.283	0.653	2.199	0.052
Right knee flexor strength (kg)	1.010	0.794	0.630	1.273	0.232
Left knee flexor strength (kg)	−0.609	0.679	−0.423	−0.897	0.391
Right ankle extensor strength (kg)	−1.022	0.762	−0.601	−10.340	0.210
Left ankle extensor strength (kg)	0.709	0.627	0.446	1.130	0.285
Right ankle flexor strength (kg)	0.330	0.461	0.249	0.715	0.491
Left ankle flexor strength (kg)	−0.042	0.556	−0.022	−0.075	0.941
Right handgrip strength (kg)	−0.149	0.250	−0.248	−0.597	0.564
Left handgrip strength (kg)	0.050	0.262	0.069	0.189	0.854
Stepwise method					
(Constant)	5.028	2.518		1.997	0.061
Left knee extensor strength (kg)	0.714	0.148	0.750	4.810	0.000

**Table 7:** Association of the strength of the left knee extensor muscles with the strength of other muscle groups (linear regression analysis, Enter method + stepwise method).

Indicators	B	Std. Error	Beta	t	Sig.
Enter method					
(Constant)	−8.714	5.568		−1.565	0.149
Right knee extensor strength (kg)	−0.232	0.783	−0.138	−0.297	0.773
Right knee flexor strength (kg)	0.221	0.644	0.146	0.343	0.739
Left knee flexor strength (kg)	0.042	0.760	0.024	0.055	0.957
Right ankle extensor strength (kg)	0.077	0.611	0.046	0.126	0.902
Left ankle extensor strength (kg)	0.258	0.427	0.185	0.604	0.559
Right ankle flexor strength (kg)	0.326	0.501	0.166	0.652	0.529
Left ankle flexor strength (kg)	0.203	0.224	0.323	0.907	0.386
Right handgrip strength (kg)	0.050	0.241	0.066	0.206	0.841
Left handgrip strength (kg)	0.524	0.238	0.499	2.199	0.052
Stepwise method					
(Constant)	−5.871	3.243		−1.811	0.089
Right knee extensor strength (kg)	0.578	0.146	0.550	3.956	0.001
Right handgrip strength (kg)	0.226	0.077	0.358	2.924	0.010
Right ankle flexor strength (kg)	0.412	0.193	0.296	2.137	0.048

## Data Availability

The datasets used and/or analyzed during the current study are available from the corresponding author upon reasonable request.
